# Clinical and demographic characteristics of male patients with Brucellar epididymo-orchitis: a retrospective cohort study from an endemic region

**DOI:** 10.3389/fmicb.2025.1673906

**Published:** 2025-10-23

**Authors:** Elad Mazor, Rozalia Smolyakov, Itai Hazan, Igor Yusim, Victor Novack, Haim Herzberg, Nicola J. Mabjeesh, Yarden Zohar

**Affiliations:** ^1^Department of Urology, Faculty of Health Science, Soroka University Medical Center, Ben-Gurion University of Negev, Be’er Sheva, Israel; ^2^Infectious Diseases Unit, Faculty of Health Science, Soroka University Medical Center, Ben-Gurion University of the Negev, Be’er Sheva, Israel; ^3^IBM Cybersecurity Center of Excellence, Be’er Sheva, Israel; ^4^Department of Software and Information Systems Engineering, Ben-Gurion University of the Negev, Be’er Sheva, Israel; ^5^Soroka Clinical Research Center, Faculty of Health Sciences, Soroka University Medical Center, Ben-Gurion University of the Negev, Be’er Sheva, Israel

**Keywords:** endemic brucella, brucellosis epididymo-orchitis, genitourinary brucella, brucellosis, male reproductive health, testicular infection

## Abstract

**Background:**

Brucellar epididymo-orchitis (BEO) is an under-recognized complication of brucellosis, which can have long-term consequences. This study aimed to identify clinical and demographic factors associated with BEO among male patients in a highly endemic population.

**Methods:**

We conducted a retrospective analysis of patients diagnosed with brucellosis confirmed by positive serological assays at our medical center. The demographic and clinical characteristics of patients with BEO were compared with those of patients with brucellosis but without epididymo-orchitis (EO).

**Results:**

Between 2001 and 2019, 2,422 individuals presented with brucellosis. Of these, 39 (1.6%) had BEO, and 2,383 had non-EO brucellosis. Most patients in both groups were of the Bedouin ethnicity (90%–94%). A comparison of age, BMI, and number of children revealed statistically significant differences. To minimize bias, a 1:3 matched comparison was performed between 117 non-EO brucellosis patients and 39 BEO patients. This comparison showed that patients with BEO had a significantly lower number of children (median of 2 vs. 6). In terms of laboratory findings, patients with BEO had a significantly higher C-reactive protein (CRP) level (median, 11 vs. 1.8). No differences were found in environmental risk factors, such as smoking.

**Conclusion:**

BEO patients were older, had higher BMI and inflammatory markers, and reported fewer children compared to non-EO brucellosis patients. These findings may reflect delayed diagnosis or chronicity, though reproductive implications remain speculative and warrant prospective evaluation.

## Introduction

Brucellosis is an endemic zoonotic disease caused by Brucella species, which are facultative intracellular gram-negative coccobacilli (Qureshi et al., 2023). The global distribution of brucellosis is uneven, with the highest reported incidence rates in Africa and Asia. Sub-Saharan Africa and the Middle East are well-known endemic regions, where a high prevalence is attributed to a combination of environmental, economic, and cultural factors (Elbehiry et al., 2023).

Brucellosis infections are most commonly observed in rural areas and are frequently caused by consumption of unpasteurized dairy products. Occupational exposure is another significant risk factor, with infection resulting from direct contact with animal secretions, inhalation of contaminated aerosols, or conjunctival contamination (Lai et al., 2021). Brucellosis is endemic in Israel, particularly among the Bedouin population in the Negev, with an incidence rate of 41 cases per 100,000 individuals in 2012 (Weinberger et al., 2024). Of note, the incidence rate is reported as doubled for males relative to females in every age group (Laine et al., 2023).

Brucellosis manifests ubiquity with multi-organ involvement and may become a chronic illness when misdiagnosed. Prevention is particularly challenging due to the animal restrictions required to control the infection route, especially in regions with illegal cross-border trade. Notably, the mortality is relatively low (Weinberger et al., 2024).

Genitourinary (GU) disease is the second most common focal organ disease, following the locomotor system (Jin et al., 2023). Approximately 2%–20% of GU brucellosis manifests as a single-organ involvement and may present as: nephritis, epididymo-orchitis (EO), prostatitis, or cystitis. Unilateral Brucellar epididymo-orchitis (BEO) is more common, whereas severe forms may progress into abscess (Jin et al., 2023).

In this study, we compared patients with focal brucellosis infection of the testis (the BEO group) and patients with non-GU brucellosis infection (non-EO brucellosis) to identify clinical and demographic factors associated with BEO among male patients in the highly endemic population of the Negev region.

## Methods

With the approval of the institutional ethics committee of Soroka University Medical Center (protocol code 0245-19-SOR), we retrospectively collected data from the medical records of patients diagnosed with Brucella infection at our institute between 2000 and 2019. Diagnosis of brucellosis was established either by a positive blood culture or by serological testing using the Brucellacapt^®^ immunocapture agglutination test (Vircell, Spain). A titer of ≥ 1:160 was considered diagnostic in accordance with our institutional protocol. Brucellacapt^®^ detects total anti-Brucella antibodies and has been reported to have a sensitivity of 97.3% and a specificity of 97.1% (Di Bonaventura et al., 2021; Casanova et al., 2009). We included all male patients with confirmed brucellosis who were hospitalized, irrespective of their disease manifestation. Female patients were excluded.

In line with standard clinical practice, only patients presenting with GU symptoms underwent a genital physical examination. All patients with suspected involvement subsequently underwent ultrasound (US) to confirm epididymal and/or testicular involvement. All 39 patients with BEO presented with acute symptoms, had a positive genital examination, and were confirmed by US. This approach ensured that all clinically significant cases of BEO were captured, reflecting real-world patient management in an endemic population. Data on additional features such as laterality, abscess formation, or severity were not consistently documented in earlier records; therefore, were not included.

**Figure 1:**
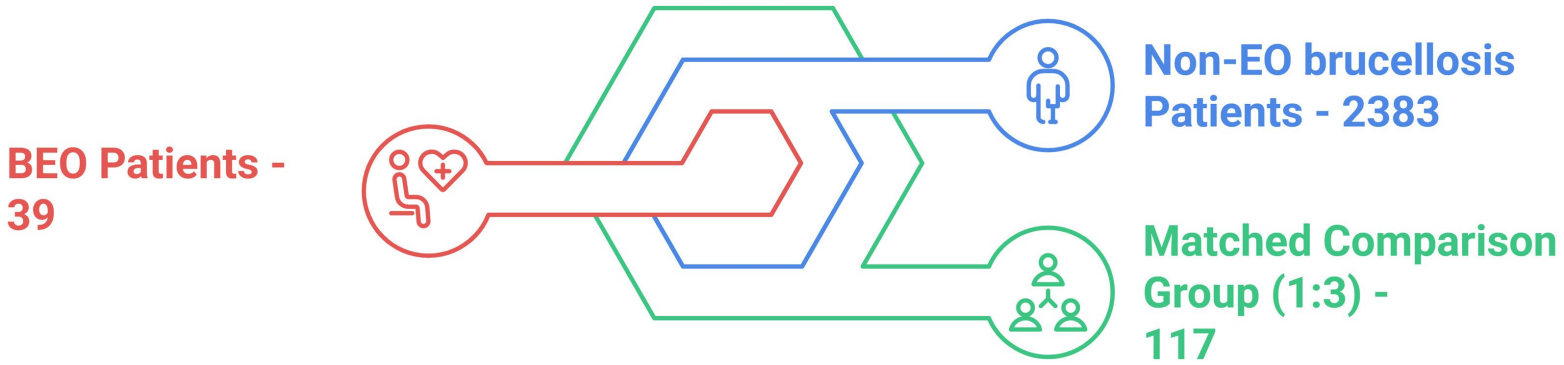
Brucellosis patients analysis. BEO, Brucellar epididymo-orchitis; EO, epididymo-orchitis.

Overall, data was collected from the medical records of 2,422 male individuals with confirmed brucellar infection. We detected only 39 cases of testicular involvement, presented as orchitis, epididymitis, or EO. Clinical, demographic, and laboratory data were compared across all ages. Data was analyzed using the “*R*” software (version 4.2.0), and define *p*-value threshold (< 0.05). Continuous variables were compared using the independent *t*-test or Mann-Whitney *U* test (Wilcoxon rank-sum test), depending on data distribution. Categorical variables were analyzed using Pearson’s chi-squared test or Fisher’s exact test, as appropriate. We applied 1:3 matching to account for imbalances between groups (Figure 1).

## Results

A total of 2,422 individuals presented with brucellosis between the years 2001 and 2019 at our center. Of them, 39 (1.6%) presented with BEO, and 2,383 with non-EO brucellosis. The vast majority of patients in both groups were of the Bedouin ethnicity (90% BEO vs. 94% with non-EO brucellosis). We found a significant difference in the mean age of BEO patients compared to non-EO brucellosis patients (32 ± 14 vs. 24 ± 18 years, respectively; *p* < 0.001) (Table 1). Sixty-two percent of BEO patients were older than 25 years compared to 37% in the non-EO brucellosis group. Interestingly, none of the BEO patients were younger than 15 years of age. The number of children was significantly lower in the BEO group (median, 2 vs. 5; *p* < 0.001). In addition, BMI was significantly higher in the BEO group (24 vs. 21, *p* = 0.003). No other parameters, including smoking status and background comorbidities, were significantly different between the groups.

**Table 1:** Demographic data of Brucella positive patients.

Characteristic	Case, *N* = 39	Control, *N* = 2,344	*p*-value^a^
Age, yrs			< 0.001
Mean ± SD (*N*)	32 ± 14 (39)	24 ± 18 (2,338)	
Median (IQR)	29 (20, 38)	17 (12, 35)	
Range	15, 64	0, 93	
Age above 25 yrs, *n*/*N* (%)	24/39 (62%)	872/2,338 (37%)	0.002
Ethnicity, *n*/*N* (%)			0.3
Bedouin	35/39 (90%)	2,191/2,342 (94%)	
Jewish	4/39 (10%)	151/2,342 (6.4%)	
Number of children			< 0.001
Mean ± SD (*N*)	3.4 ± 4.2 (39)	6.5 ± 6.1 (519)	
Median (IQR)	2.0 (0.0, 5.0)	5.0 (2.0, 10.0)	
Range	0.0, 16.0	0.0, 37.0	
BMI, mean ± SD (*N*)	24.0 ± 4.2 (23)	21.0 ± 5.3 (886)	0.003
Smoking, *n*/*N* (%)	13/30 (43%)	513/1,546 (33%)	0.2
CCI, mean ± SD (*N*)	0.26 ± 0.75 (39)	0.21 ± 0.79 (2,344)	0.5

CCI, Charlson comorbidity index; IQR, interquartile; SD, standard deviation.

a Wilcoxon rank sum test; Pearson’s Chi-squared test; Fisher’s exact test.

Based on the initial comparison differences between BEO and non-EO brucellosis individuals (*n* = 39 vs. *n* = 2,383), we matched the non-EO brucellosis group in terms of age and BMI to avoid statistical biases. To improve statistical power, we matched the groups in a 1:3 ratio; thus, the matched comparison was between 117 individuals with non-EO brucellosis and 39 patients with BEO (Table 2). Comparison between the matched groups revealed that the number of children in the BEO group was significantly lower than that in the non-EO brucellosis group, with a median of 2 vs. 6. Regarding inflammatory serum markers, patients who presented with BEO had C-reactive protein (CRP) levels (mg/dL) of 11 vs. 1.8 for the non-EO brucellosis patients (Table 3). However, the mean hospitalization days was not significantly different between the BEO and non-EO brucellosis groups (8.1 ± 5.2 vs. 6.6 ± 4.8, *p* = 0.14, respectively). There were no statistical differences between the groups in terms of environmental risk factors, such as smoking, when the matched groups were compared.

**Table 2:** Demographic data of Brucella positive patients after 1:3 matching.

Characteristic	Case, *N* = 39	Control, *N* = 117	*p*-value^a^
Age, yrs			> 0.9
Mean ± SD (*N*)	32 ± 14 (39)	32 ± 14 (117)	
Median (IQR)	29 (20, 38)	29 (20, 38)	
Range	15, 64	15, 64	
Age above 25 yrs, *n*/*N* (%)	24/39 (62%)	72/117 (62%)	> 0.9
Ethnicity, *n*/*N* (%)			0.8
Bedouin	35/39 (90%)	99/115 (86%)	
Jewish	4/39 (10%)	16/115 (14%)	
Number of children			0.008
Mean ± SD (*N*)	3.4 ± 4.2 (39)	6.2 ± 4.8 (31)	
Median (IQR)	2.0 (0.0, 5.0)	6.0 (2.5, 10.0)	
Range	0.0, 16.0	0.0, 15.0	
BMI, mean ± SD (*N*)	24.0 ± 4.2 (23)	23.4 ± 3.8 (51)	0.7
Smoking, *n*/*N* (%)	13/30 (43%)	40/96 (42%)	0.9
CCI, mean ± SD (*N*)	0.26 ± 0.75 (39)	0.19 ± 0.72 (117)	0.3

CCI, Charlson comorbidity index; IQR, interquartile; SD, standard deviation.

a Wilcoxon rank sum test; Pearson’s Chi-squared test; Fisher’s exact test.

**Table 3:** Outcomes of Brucella-positive patients following 1:3 matching.

Characteristic	Case, *N* = 39	Control, *N* = 117	*p*-value^a^
C-reactive protein (mg/dL)			< 0.001
Mean ± SD (*N*)	10.9 ± 8.5 (17)	3.8 ± 4.8 (30)	
Median (IQR)	11.0 (4.4, 13.8)	1.8 (0.5, 4.8)	
Range	0.3, 37.9	0.1, 18.6	
WBC			0.002
Mean ± SD (*N*)	8.19 ± 2.80 (31)	6.48 ± 2.27 (91)	
Median (IQR)	8.31 (6.31, 9.47)	6.14 (5.04, 7.66)	
Range	3.69, 15.17	2.31, 15.64	
NEUT. abs (10^3^/μL)			< 0.001
Mean ± SD (*N*)	5.18 ± 2.48 (31)	3.36 ± 1.79 (91)	
Median (IQR)	5.40 (3.12, 6.36)	3.10 (2.31, 3.86)	
Range	1.41, 11.82	1.06, 13.34	
NEUT. abs/LYMP. abs			< 0.001
Mean ± SD (*N*)	2.62 ± 1.35 (31)	1.75 ± 1.46 (91)	
Median (IQR)	2.46 (1.49, 3.76)	1.30 (0.94, 1.83)	
Range	0.62, 5.51	0.34, 8.55	
Hospital days, mean ± SD (*N*)	8.1 ± 5.2 (24)	6.6 ± 4.8 (33)	0.14

Abs, absolute; IQR, interquartile; LYMPH, lymphocytes; NEUT, neutrophils; SD, standard deviation.

a Wilcoxon rank sum test.

## Discussion

Brucellosis is a zoonotic infection that should be considered in endemic areas. The disease can be diagnosed in the acute (0–2 months), subacute (2–12 months), or chronic (> 12 months) phases, typically presenting with systemic symptoms, although isolated organ involvement is also possible (Jin et al., 2023). GU involvement is the second most common focal manifestation of brucellosis, following osteoarticular involvement (Dabaja-Younis et al., 2023). Brucellar organotropism is largely attributed to its affinity for the reticuloendothelial system and is confirmed to occur primarily via hematogenous spread (Celik et al., 2023). Nevertheless, the mechanisms underlying genital tropism are not fully understood but appear to involve tissue-specific metabolic cues. Studies suggest that erythritol, abundant in the placenta, and fructose, predominant in epididymal and seminal fluids, serve as preferred carbon sources that support Brucella survival (Letesson et al., 2017; Roop et al., 2021). The reported incidence of BEO ranges from 2 to 20%, with most cases presenting in the acute phase, which is often preceded by fever (Alarbid et al., 2023).

Based on our analysis, primary risk factors for acute BEO infection include an average age in the third decade of life, with a higher BMI. Although this study was conducted in an endemic area, over the past two decades, only 1.6% of 2,422 diagnosed male brucellosis cases have been identified as BEO. Most of these cases (90%–94%) were of Bedouin ethnicity, as previously reported in local official records (Weinberger et al., 2024; Megged et al., 2016).

The higher exposure rate among individuals of Bedouin origin could be attributed to several cultural and lifestyle factors. Understanding this trend requires familiarity with Bedouin customs, including polygamy, consumption of raw food and unpasteurized milk, and close contact with livestock (Weinberger et al., 2024). Additionally, a significant proportion of young Bedouins work as shepherds, further increasing the risk of infection (Megged et al., 2016). This population also tends to delay seeking medical care, leading to delayed diagnoses and a higher likelihood of disease flares, progression, severe manifestations, and chronic infection (Lai et al., 2021; Weinberger et al., 2024; Megged et al., 2016; Dean et al., 2012; Batirel et al., 2020). Indeed, BEO is often considered a complication of chronic brucellosis (Dabaja-Younis et al., 2023).

Regarding age distribution, 62% of individuals in the BEO group were over the age of 25 years, and none were younger than 15 years, suggesting a possible association with sexual activity. Previous reports have suggested the sexual transmission of Brucella (Meltzer et al., 2010; Tuon et al., 2017). This is further supported by the observed age range of 15–64 years in the BEO group compared with 0–93 years in the non-EO brucellosis group. Notably, the BEO group consisted exclusively of individuals within a sexually active age range, whereas the non-EO brucellosis group spanned all ages.

In patients under 35 years of age, EO is often caused by sexually transmitted infections such as *Chlamydia trachomatis* and *Neisseria Gonorrhea* (Workowski et al., 2021). Treatment is frequently guided by clinical presentation and anamnesis, and may be initiated empirically with a 10- to 14-day course of doxycycline, even in the absence of microbiological confirmation (Workowski et al., 2021; Hazra et al., 2022).

However, while doxycycline is also a primary treatment for brucellosis, a minimum of 6 weeks is required for effective therapy (Celik et al., 2023). This raises concerns regarding under-diagnosis and misdiagnosis of BEO in endemic populations, as Brucella is less commonly suspected. Patients may be undertreated if cultures are not performed, increasing the risk of progression to the chronic phase (Meltzer et al., 2010; Tuon et al., 2017; Workowski et al., 2021; Li et al., 2020).

Furthermore, based on the cohort analysis correlating BEO presentation with the number of children per patient, individuals diagnosed with BEO tended to have fewer children. This may be partially attributed to the chronic phase of the disease or relapse during which the infection has already compromised fertility.

The association between Brucella infection and male sexual dysfunction has been previously explored (Celik et al., 2023; Meltzer et al., 2010; Li et al., 2020; Safwat et al., 2018). Several mechanisms have been identified through which Brucella can impair virility (Yu et al., 2022). One of the most well-documented and direct mechanisms involves bacterial invasion of the testicular tissue (Wang et al., 2021). Brucella is a facultative intracellular pathogen capable of evading macrophages and circumventing targeted immunity, allowing it to persist in the host tissues that inducing chronic inflammation, and promoting fibrosis (Schuppe et al., 2008). This inflammatory response can lead to testicular atrophy, which is often observed during follow-up, whereas bacterial presence in the semen may be detectable at the time of diagnosis (Celik et al., 2023; Akinci et al., 2006). Notably, chronic BEO may progress to testicular abscess formation and necrosis (Celik et al., 2023; Jin et al., 2023), necessitating surgical intervention including orchiectomy (Jin et al., 2023). In such cases, infertility would be a prospective consequence rather than a pre-existing one (Schuppe et al., 2008; Zhang et al., 2022).

Another mechanism contributing to sexual dysfunction in BEO involves hormonal dysregulation and associated impotence, which are interrelated (Yu et al., 2022). Although BEO typically affects only one testis, infertility remains a significant concern and often persists even after disease resolution (Yu et al., 2022; Fomichova et al., 2025; Abroudi et al., 2025). Notably, 70% of the patients with BEO have reported erectile dysfunction (ED) (Safwat et al., 2018). In a 10-year multicenter series, Celik et al. (2023) followed 190 patients with brucellosis-related testicular involvement, of whom spermiogram data were available for only 6.8%; among these, oligozoospermia and azoospermia were observed in 41.7% and 8.3%, respectively.

Brucella spp. has been detected in female genital tract tissues, and rare instances of human-to-human transmission through sexual contact have been reported (Meltzer et al., 2010; Tuon et al., 2017; Li et al., 2020). A recent systematic review identified 10 probable cases of sexual transmission, two of which were confirmed by culture or PCR of semen. In all reported cases, infection occurred sequentially between partners, and alternative transmission routes were excluded (Tuon et al., 2017). Although uncommon, these findings provide microbiological support for sexual transmission and underscore the need for further investigation into this route of infection.

In addition to direct testicular involvement, disruption of the hypothalamic–pituitary-gonadal axis plays a role in infertility as well (Rodríguez et al., 2019; Safdari, 2025). Affected males with chronic BEO have been found to exhibit low testosterone levels, which may be attributed to direct Leydig cell destruction caused by localized testicular bacterial inflammation (Yu et al., 2022; Zhou et al., 2020). Cytokine-mediated inflammation contributes to this dysfunction, particularly through elevated hematogenous secretion of tumor necrosis factor-alpha (TNF-*α*) (Xu et al., 2024). In a study by Safwat et al. (2018), testosterone supplementation improved ED, especially in younger patients, likely owing to its inhibitory effect on TNFα. Moreover, Brucella-induced breach of the blood-testis barrier can promote autoimmunity, with chronic immune-mediated responses in the affected testis potentially impairing the contralateral testis via autoantibodies (Akinci et al., 2006; Xu et al., 2024; Goericke-Pesch et al., 2022). In addition, direct oxidative stress to Sertoli cells during infection may compromise spermatogenesis and sperm quality (Fomichova et al., 2025).

Collectively, these mechanisms highlight how BEO can cause persistent infertility and sexual dysfunction even after resolution of the acute infection (Yu et al., 2022; Abroudi et al., 2025).

Other significant findings include markedly higher inflammatory marker values in the BEO group compared with the non-EO brucellosis group (CRP: 11 mg/dL vs. 1.8 mg/dL, *p* < 0.001; Absolute Neutrophil Count (ANC): 5.4 × 10^3^/μL vs. 3.1 × 10^3^/μL, *p* < 0.001; Neutrophil-to-Lymphocyte Ratio: 2.4 vs. 1.3, *p* < 0.001). This may reflect a predisposition of testicular tissue to abscess formation and a more pronounced local immune response (Hamoda et al., 2023).

Overall, the evidence indicates that BEO can compromise male reproductive function through multiple, interrelated mechanisms, including direct testicular damage, hormonal dysregulation, oxidative stress to Sertoli cells, and immune-mediated impairment of both testes (Yu et al., 2022). These pathophysiological processes may collectively contribute to persistent infertility and sexual dysfunction, even after resolution of the acute infection (Abroudi et al., 2025). Although BEO patients in this study had fewer children, causality cannot be definitively established due to the retrospective design, the lack of systematic fertility assessments (including semen analysis and hormonal profiling), and the absence of data on the timing of childbirth. Nonetheless, these findings underscore the potential long-term reproductive consequences of BEO and highlight the need for prospective studies to quantify fertility outcomes and guide early interventions.

Primary limitations of this study include its retrospective design and the lack of critical data needed to fully confirm our hypotheses. These limitations should be considered when interpreting our findings, and prospective studies are warranted to address these gaps.

Future studies should prospectively collect data on semen analyses, follicle-stimulating hormone, luteinizing hormone, and testosterone levels. Additionally, investigating the presence of Brucella bacilli in body tissues and fluids may provide further insights into the pathogen’s mechanism of action.

Nonetheless, to our knowledge, this is the first cohort study reporting the number of children among patients diagnosed with BEO, suggesting a possible link to unresolved, chronic, or recurrent Brucella infections, particularly in endemic populations. Future research to validate this hypothesis should include female patients presenting with brucellar vaginitis, salpingitis, or cervicitis, to assess age distribution in relation to sexual activity and fertility outcomes.

## Conclusion

Our findings suggest that physicians should have a higher index of suspicion for BEO in sexually active individuals over 15 years of age, particularly in those with a high BMI, history of few children, and residence in an endemic area. These clinical indicators, along with laboratory findings such as elevated CRP, can aid in early detection.

## Data Availability

The original contributions presented in the study are included in the article/supplementary material, further inquiries can be directed to the corresponding author.
